# Computerized cognitive training for memory functions in mild cognitive impairment or dementia: a systematic review and meta-analysis

**DOI:** 10.1038/s41746-023-00987-5

**Published:** 2024-01-03

**Authors:** Aaron T. C. Chan, Roy T. F. Ip, Joshua Y. S. Tran, Joyce Y. C. Chan, Kelvin K. F. Tsoi

**Affiliations:** 1 JC School of Public Health and Primary Care, Faculty of Medicine, The Chinese University of Hong Kong, Hong Kong, China; 2 Stanley Ho Big Data Decision Analytics Research Centre, The Chinese University of Hong Kong, Hong Kong, China

**Keywords:** Rehabilitation, Dementia

## Abstract

Dementia is a common medical condition in the ageing population, and cognitive intervention is a non-pharmacologic strategy to improve cognitive functions. This meta-analysis evaluated the benefits of computerized cognitive training (CCT) on memory functions in individuals with MCI or dementia. The study was registered prospectively with PROSPERO under CRD42022363715 and received no funding. The search was conducted on MEDLINE, Embase, and PsycINFO on Sept 19, 2022, and Google Scholar on May 9, 2023, to identify randomized controlled trials that examined the effects of CCT on memory outcomes in individuals with MCI or dementia. Mean differences and standard deviations of neuropsychological assessment scores were extracted to derive standardized mean differences. Our search identified 10,678 studies, of which 35 studies were included. Among 1489 participants with MCI, CCT showed improvements in verbal memory (SMD (95%CI) = 0.55 (0.35–0.74)), visual memory (0.36 (0.12–0.60)), and working memory (0.37 (0.10–0.64)). Supervised CCT showed improvements in verbal memory (0.72 (0.45–0.98)), visual memory (0.51 (0.22–0.79)), and working memory (0.33 (0.01–0.66)). Unsupervised CCT showed improvement in verbal memory (0.21 (0.04–0.38)) only. Among 371 participants with dementia, CCT showed improvement in verbal memory (0.64 (0.02–1.27)) only. Inconsistency due to heterogeneity (as indicated by I^2^ values) is observed, which reduces our confidence in MCI outcomes to a moderate level and dementia outcomes to a low level. The results suggest that CCT is efficacious on various memory domains in individuals with MCI. Although the supervised approach showed greater effects, the unsupervised approach can improve verbal memory while allowing users to receive CCT at home without engaging as many healthcare resources.

## Introduction

Dementia is a common medical condition among the elderly in which the symptoms of cognitive impairment significantly affect social functioning and daily living^1^. While Alzheimer’s Disease (AD) is the most common type of dementia, other underlying etiologies can also cause dementia and mild cognitive impairment (MCI). These include vascular brain injury resulting from strokes or microvascular infarcts, the accumulation of Lewy bodies in the brain, and other clinical diseases like Parkinson’s Disease^2,3^. It was estimated that 57.4 million people were living with dementia in 2019 and the number will reach 152.8 million by 2050^2^. The prevalence of dementia was estimated to be around 5-7% in the ageing population^3^. According to the World Health Organization, the estimated global cost of dementia was US $1.3 trillion in 2019, and it is the seventh leading cause of death worldwide^4^. Meanwhile, MCI is a prodromal stage in which individuals suffer from cognitive impairment but remain capable of maintaining instrumental activities of daily living^5,6^. The prevalence of MCI in the general population also increases with age, ranging from 6.7% in individuals aged 60-64 to as much as 25.2% in individuals aged 80 or above^5^. It was estimated that in the individuals aged 60 years or older, those with MCI were twice as likely than those with normal cognition to have cerebral amyloid-β aggregation, which is a key indicator of AD pathology^7^.

Memory deficit is one of the most observable and common symptoms among individuals with MCI or early dementia that disturbs the quality of life. An epidemiologic study suggested that the prevalence of amnestic MCI in the elderly population was more than double compared to the non-amnestic type^8^. Memory deficit is also considered to be the clinical hallmark of AD^5^ and individuals with AD often show deficits in episodic memory at very early stages of the disease^9^. Individuals with amnestic MCI have been found to have a significantly higher risk of AD and amyloid-β aggregation compared to those with the non-amnestic type^7^. The elderly with subjective memory complaints (SMC) are shown to have a higher risk of dementia than the individuals without SMC^10^. Memory decline is also found among the elderly to be independently associated with the ability to carry out instrumental activities of daily living^11,12^.

Cognitive intervention is generally regarded as an effective non-pharmacologic strategy to mitigate risks in neurodegeneration. The 2018 American Academy of Neurology guideline posits that cognitive intervention may be effective in improving cognitive function that clinicians may recommend it to people with MCI^5^. The underlying mechanism is proposedly through the enhancement of cognitive reserve, which improves resilience against neurodegeneration^13^. In addition, MRI-based studies found that cognitive training changed the resting brain state by improving cerebral blood flow, connectivity in brain networks and white matter integrity in healthy seniors, suggesting one of the benefits of cognitive training is induced through enhancing neuroplasticity^14^. Non-pharmacologic management for MCI is important as currently there is no disease-modifying treatment or FDA-approved medications for MCI^5,15^. Conventionally, cognitive interventions for MCI were delivered in a face-to-face, paper-and-pencil setting by trained professionals, but nowadays cognitive interventions are also available in computerized versions. Computerized cognitive training (CCT) can be more easily incorporated with technological features, such as adaptive difficulties according to real-time performance to keep the tasks engaging and challenging. Immersive virtual reality (VR) and internet connectivity can also be incorporated into the training regime which enriches the experience beyond the traditional boundary in the paper-and-pencil setting. Compared to traditional paper-and-pencil cognitive training, the administration of CCT is guided through a computerized process, reducing the workload and skills required of the trainers.

Although CCT processes are computerized, most CCT interventions are designed to be administered under the face-to-face supervision of a trained professional, such as a clinician or a therapist, to ensure adherence and respond to technical difficulties. By contrast, unsupervised CCT fully utilizes the automation element and allows the subjects to administer the CCT at home by themselves or caregivers without real-time supervision by professionals, so it saves healthcare resources. Despite the significance of unsupervised CCT, none of the previous meta-analyses on CCT^16,17^ has reported the difference in efficacies between supervised CCT and unsupervised CCT in people with MCI or dementia. Therefore, this study aims to (1) review the latest studies on CCT, (2) provide an updated assessment of its benefits on memory function in people with MCI or dementia, and (3) compare the effectiveness of unsupervised and supervised CCT.

## Results

### Study selection

**Fig. 1:**
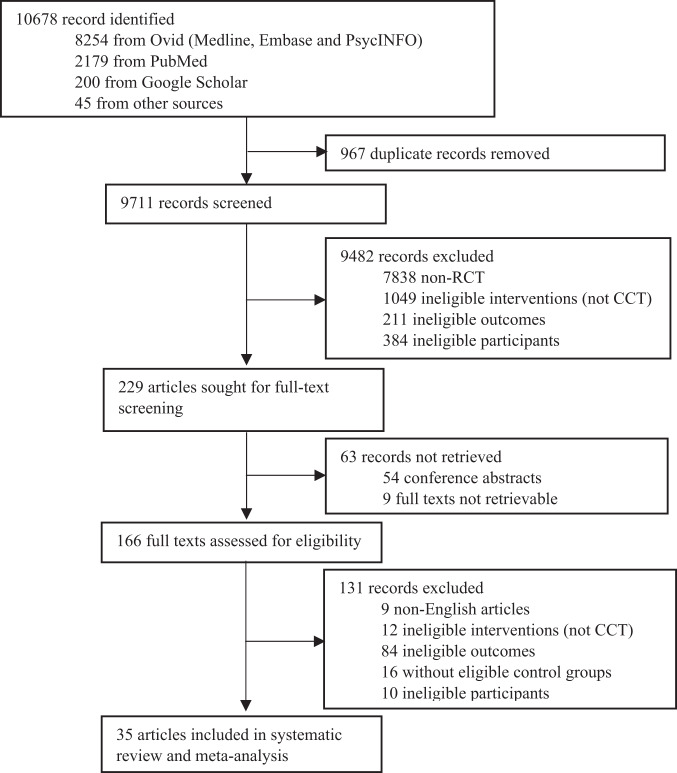
PRISMA flow diagram of included studies.

A total of 10,678 literature records were identified from the databases. After removal of duplicate records, titles and abstracts screening was conducted on 9711 records. Full-text screening for eligibility assessment was conducted on 229 records and finally 35 articles were included in this systematic review (Fig. 1). Seven articles included participants with dementia only and 26 articles included participants with MCI only. Two articles reported data on the participants with MCI and those with dementia separately, so each of these two articles was separated into two comparisons (i.e., MCI and dementia respectively) for analysis^18,19^. As a result, 28 studies with 1489 participants with MCI and 9 studies with 371 participants with dementia were included.

### Characteristics of studies

Among the 35 eligible studies with 37 comparisons for the interventions of CCT, nine of the studies used home-based, unsupervised CCT while the remaining 26 studies used supervised CCT (Table 1). The sample sizes of individual studies ranged from 13 to 141 participants with mean ages between 44 and 81 years. Baseline cognitive assessment scores among the participants ranged from 16.4 to 26.2 (MoCA) and 16.1 to 28.1 (MMSE), so participants were mainly in the early stage of dementia or MCI. Nine of the 35 studies were at high risk of bias and the remaining 26 studies either raised some concerns or were at low risk of bias (Supplementary Table 1). Studies delivered CCT via different devices, including traditional desktop computers (74.3%), touch-screen computers or tablets (20%), and computers equipped with immersive virtual reality (VR) functions (5.7%). The intensity of CCT training was mainly one to two hours per week. The duration of training ranged from two weeks to six months. Amongst the studies that have reported adherence data, the completion rates ranged between 64.1% and 100%. Nine out of 11 studies on supervised CCT and two out of nine studies on unsupervised CCT reported 100% of training completion. A detailed summary of adherence data can be found in Supplementary Fig. 1.

**Table 1:** Characteristics of included studies on computerized cognitive training.

Study ID (Year)	Memory Domains Reported	CCT Delivery Method	Device	Control Group	Study Location	Total Training Hours	Duration (Weeks)	Training Hours per Week	Types of Impairment	Sample Size		Mean Age	Gender (% Male)	Baseline Cognition	
										CCT	Control			MMSE	MoCA
Heiss^43^	Verbal/Working	Supervised	Traditional Computer	UC	Germany	48	24	2.0	Dementia	18	17	66	54%	20.4	
Tarraga^44^	Verbal	Supervised	Traditional Computer	UC	Spain	26	24	1.1	Dementia	15	16	77	23%	21.6	
Barnes^45^	Verbal/Working	Unsupervised	Traditional Computer	CA	USA	50	6	8.3	MCI	22	25	74	60%	NA	
Finn^46^	Working/Visual	Unsupervised	Traditional Computer	UC	Australia	NA	11	NA	MCI	8	8	73	50%	27.8	
Boller^47^	Verbal/Working/Visual	Supervised	Traditional Computer	UC	France	6	2	3.0	Dementia	12	12	81	38%	24.9	
Herrera^48^	Verbal/Working/Visual	Supervised	Traditional Computer	PAP-UA	France	24	12	2.0	MCI	11	11	77	50%	27.3	
Lee^49^	Verbal	Supervised	Touch-screen Computer	UC	Hong Kong	6	6	1.0	Dementia	7	6	78	32%	16.1	
Fiatarone^50^	Verbal/ Visual	Supervised	Touch-screen Computer	CA	Australia	60	24	2.5	MCI	24	27	70	32%	27	
Tarnanas^51^	Verbal/Working/Visual	Supervised	Traditional Computer	UC	Greece	60	20	3.0	MCI	39	34	70	41%	26.5	
Finn^52^	Verbal/Working	Supervised	Traditional Computer	UC	Australia	NA	4	NA	MCI	12	12	75	71%	28.1	
Barban^18^	Verbal	Supervised	Touch-screen Computer	UC	Italy, Greece, Norway, and Spain	24	12	2.0	MCI	46	60	74	53%	27.8	
									Dementia	42	39	77	30%	23.4	
Cavallo^22^	Verbal/Working	Supervised	Traditional Computer	CA	Italy	18	12	1.5	Dementia	40	40	76	57%	22.9	
Gooding^53^	Verbal/Visual	Supervised	Traditional Computer	CA	USA	30	16	1.9	MCI	31	20	76	58%	NA	
Hyer^54^	Working	Supervised	Traditional Computer	CA	USA	17	5-7	2.4-3.4	MCI	34	34	75	47%	NA	
Lin^55^	Working	Unsupervised	Traditional Computer	CA	China	15	6	2.5	MCI	10	11	73	52%		25
Hagovska^56^	Verbal	Supervised	Traditional Computer	PAP-CT	Slovakia	10	10	1.0	MCI	30	30	68	48%	25.3	
Han^57^	Verbal	Unsupervised	Tablet	UC	Korea	4	4	1.0	MCI	43	42	74	54%	25.1	
Savulich^58^	Visual	Supervised	Tablet	UC	UK	8	4	2.0	MCI	21	21	76	60%	26.7	
De Luca^59^	Verbal/Working	Supervised	Traditional Computer	PAP-CT	Italy	18	8	2.3	Dementia^a^	20	15	44	55%	23.2	
Nousia^23^	Verbal/Working	Supervised	Traditional Computer	UC	Greece	15	15	1.0	Dementia	25	25	76	28%		16.4
Bernini^60^	Verbal/Working/Visual	Supervised	Traditional Computer	UC	Italy	9	4	2.3	MCI^a^	17	18	70	35%	25.3	
Li^61^	Verbal/Visual	Unsupervised	Traditional Computer	UC	China	48-64	24	2.0-3.0	MCI	78	63	71	NA	28	
Poptsi^62^	Verbal/Working	Supervised	Traditional Computer	PAP-CT	Greece	48	24	2.0	MCI	14	18	69	31%	28	
Tang^63^	Verbal/Working	Unsupervised	Traditional Computer	CA	China	18	7	2.6	MCI^a^	30	30	64	67%		21.6
Yang^64^	Verbal/Working/Visual	Supervised	Traditional Computer	CA	Taiwan	27	12	2.3	MCI	33	33	79	21%	27	23.9
Maneti^65^	Verbal	Unsupervised	Tablet	PAP-CT	Italy	36	4	9.0	MCI	18	17	77	49%	NA	
Park^66^	Working	Supervised	Computer with VR	UC	Korea	12	12	1.0	MCI	10	11	71	33%	25.8	
Bernini^67^	Working	Supervised	Traditional Computer	PAP-CT	Italy	9	3	3.0	MCI^a^	18	12	71	73%	25.3	19.6
Callisaya^68^	Verbal	Unsupervised	Tablet	UC	Australia	48	24	2.0	MCI	44	44	73	42%		26.2
Kang^69^	Verbal/Working	Supervised	Computer with VR	UC	Korea	4	4	1.0	MCI	23	18	75	29%	26.2	
Nousia^70^	Verbal/Working	Supervised	Traditional Computer	UC	Greece	30	15	2.0	MCI	25	21	72	24%		21.8
Park^71^	Verbal	Supervised	Traditional Computer	UC	Korea	18	8	2.3	MCI	28	28	72	41%	26.6	
van Balkom^19^	Verbal/Working/Visual	Unsupervised	Traditional Computer	CA	Netherlands	18	8	2.3	MCI^a^	43	40	64	66%		26.1
									Dementia^a^	9	13	62	78%		24.5
Yeh^72^	Verbal/Working	Supervised	Traditional Computer	AE	Taiwan	36	12	3.0	MCI^a^	18	18	60	72%		19.8
Wu^73^	Verbal/Visual	Supervised	Traditional Computer	UC	China	24	8	3.0	MCI	27	26	67	23%	NA	

^a^denotes studies that included participants with Parkinson’s Disease or stroke-induced dementia.

*CCT* computerized cognitive training, *MCI* mild cognitive impairment, *VR* virtual reality

Control Group: *CA* computer activities, UC usual care, *PAP-CT* paper-and-pencil cognitive training, *PAP-UA* paper-and-pencil unstructured activities.

### Effects of CCT on MCI outcomes

Overall, 1489 participants with MCI from 28 studies compared CCT for verbal episodic memory (78.5%), visual episodic memory (39.2%), and working memory (60.7%). CCT overall showed significant improvements in verbal episodic memory (SMD (95%CI) = 0.55 (0.35–0.74)), visual episodic memory (0.36 (0.12–0.60)), and working memory (0.37 (0.10–0.64)) (Table 2).

**Table 2:** Main outcomes on Computerized Cognitive Training (CCT).

Verbal Episodic			Visual Episodic		Working Memory	
	no. of Study	SMD (95%CI)	no. of Study	SMD (95%CI)	no. of Study	SMD (95%CI)
MCI						
- Supervised CCT	15	**0.72 (0.45–0.98)**	8	**0.51 (0.22–0.79)**	12	**0.33 (0.01–0.66)**
- Unsupervised CCT	7	**0.21 (0.04–0.38)**	3	0.05 (−0.20–0.31)	5	0.49 (−0.09–1.06)
Overall	22	**0.55 (0.35–0.74)**	11	**0.36 (0.12–0.60)**	17	**0.37 (0.10–0.64)**
Dementia	9	**0.64 (0.02–1.27)**	2	0.36 (−0.24–0.95)	6	0.24 (−0.28–0.76)
-without two outliers	7	0.23 (−0.02–0.49)				

*CCT* computerized cognitive training, *CI* confidence interval, *MCI* mild cognitive impairment, *SMD* standardized mean difference.

The effect sizes with statistical significance (at 95% confidence level) are highlighted in bold.

**Fig. 2:**
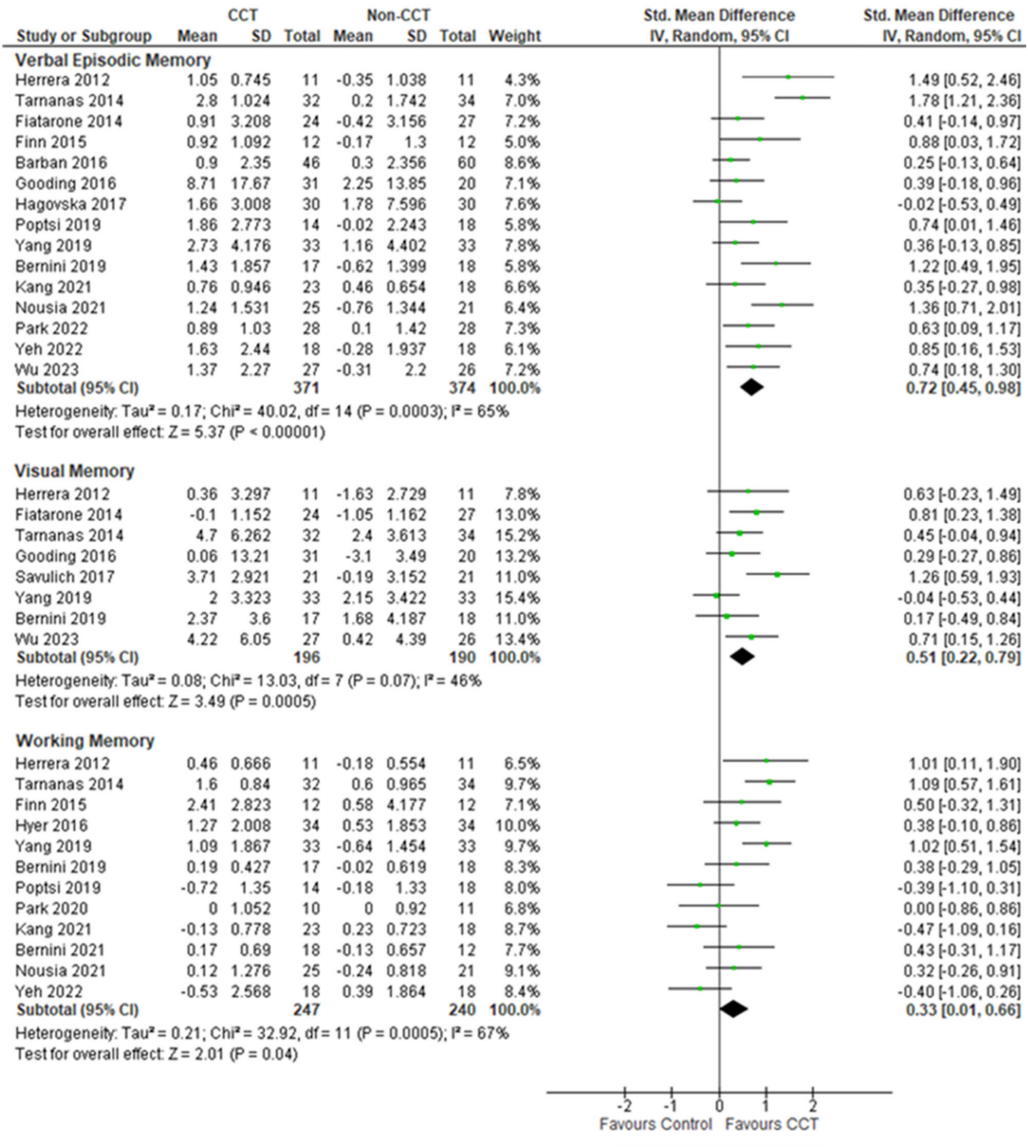
CCT (Supervised) on memory performance in individuals with MCI. CCT computerized cognitive training, CI confidence interval, MCI mild cognitive impairment, SD standard deviation.

Nineteen of these studies, with 913 participants with MCI, used supervised CCT, and they were evaluated for verbal episodic memory (78.9%), visual episodic memory (42.1%), and working memory (63.2%). Supervised CCT showed significant improvements in verbal episodic memory (SMD (95%CI) = 0.72 (0.45–0.98)), visual episodic memory (0.51 (0.22–0.79)), and working memory (0.33 (0.01–0.66)) (Fig. 2).

**Fig. 3:**
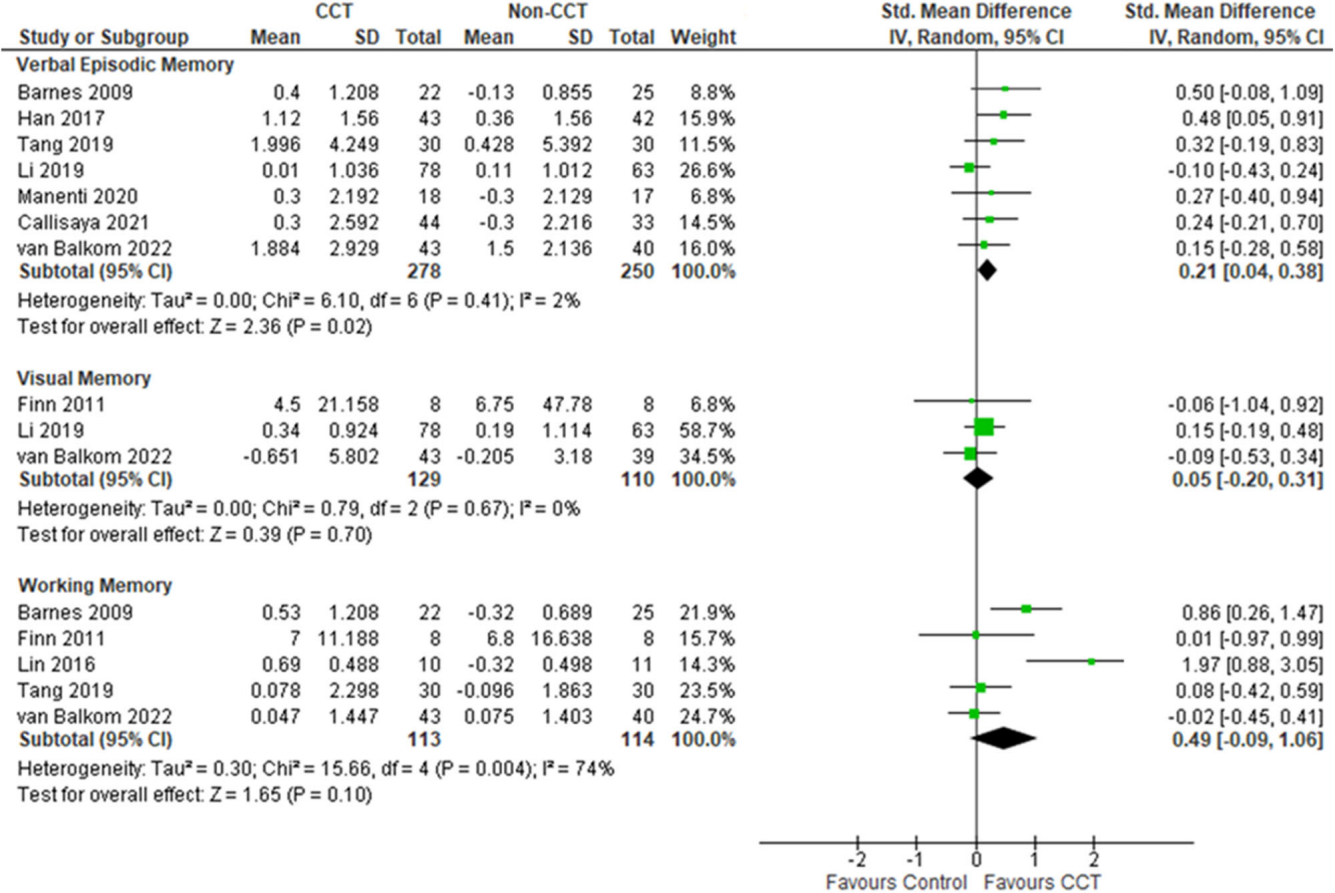
CCT (Unsupervised) on memory performance in individuals with MCI. CCT computerized cognitive training, CI confidence interval, MCI mild cognitive impairment, SD standard deviation.

The remaining nine studies, with 576 participants with MCI, used unsupervised CCT and they were evaluated for verbal episodic memory (77.8%), visual episodic memory (33.3%), and working memory (55.6%). Unsupervised CCT showed marginally improved verbal episodic memory (0.21 (0.04-0.38)), but not visual episodic memory, and working memory (Fig. 3).

### Subgroup analyses on MCI outcomes

A subgroup analysis was conducted on various durations of training time. Comparing the CCT training time for the participants with MCI, 12 studies provided CCT training for 4–18 h, eight studies provided CCT training of 19–36 h, six studies provided CCT training of >36 h and two studies did not report a total training time (Supplementary Table 2). In verbal episodic memory, CCT showed significant improvement for total training time of 4–18 h (0.40 (0.14–0.65)), 19-36 h (0.63 (0.33–0.93)) and more than 36 h (0.57 (0.04–1.10)). In visual episodic memory, CCT only showed significant improvement for a total training time of more than 36 h (0.41 (0.04–0.78)), but not for a total training time of 4–18 or 19–36 h. In working memory, CCT did not show significant improvement in each training time subgroup.

Another subgroup analysis was conducted to compare active-controlled studies with passive-controlled studies. Fourteen studies used active control, such as unstructured computer activities or paper-and-pencil cognitive activities, and the other fourteen studies offered usual care (Supplementary Table 3). In verbal episodic memory, studies that used usual care as control showed stronger benefits from CCT than those using active control (0.67 (0.33–1.01) vs 0.39 (0.20–0.58)). Otherwise, no systematic difference was observed in the visual episodic memory and working memory across different control groups.

### Effects of CCT on dementia outcomes

Among 371 participants with dementia, CCT only showed significant improvements in verbal episodic memory (0.64 (0.02–1.27)), but not visual episodic memory, and working memory (Table 2).

### Risk of bias and publication bias

Nine of the 35 studies were at high risk of bias and the remaining 26 studies either raised some concerns or were at a low risk of bias (Supplementary Table 1). Sensitivity analyses were conducted by removing the studies with a high risk of bias in each meta-analysis that had given significant results. After the removal of these high-risk-of-bias studies, all significant results retained significance except for the CCT’s effect on Verbal Memory in Dementia, of which the effect size shifted considerably from 0.64 (0.02–1.27) to 0.89 (−0.55–2.32) and became insignificant. Two outcomes (Overall CCT’s Effects on Verbal Memory in MCI and Supervised CCT’s Effects on Verbal Memory in MCI) were found to display asymmetries in funnel plots, but their effect estimates remained significant after trim-and-fill adjustments (Supplementary Table 4).

### GRADE assessment

Based on the GRADE assessment on each significant effect estimate, this study concludes that: (1) the overall CCT and supervised CCT confer benefits on verbal, visual and working memory in people with MCI, at moderate certainty; (2) the unsupervised CCT confers benefits on verbal memory in people with MCI, at moderate certainty; and (3) the overall CCT confers benefits on verbal memory in people with dementia, at low certainty. The downgrading of certainty for both MCI and dementia outcomes was due to serious inconsistency as indicated by the high I^2^ values observed. The certainty for the dementia outcome was further downgraded due to the serious risk of bias, as more than half of the included dementia studies were at high risk of bias.

## Discussion

This meta-analysis demonstrates that both supervised and unsupervised CCT improve different types of memory domains among individuals with MCI. Memory deficit is a prevalent complaint and is independently associated with a decline in quality of life, therefore, many cognitive interventions have incorporated training components of verbal, visual, and working memory. Although the supervised approach of computerized cognitive training showed the greatest benefits, the unsupervised approach can also improve verbal memory while allowing users to receive CCT at home without engagement of the healthcare professionals.

The efficacies of CCT found on different memory domains are mostly consistent with previous reports^16,17^ and demonstrate that CCT is a viable non-pharmacological intervention for people with MCI in improving memory performance. A previous meta-analysis by Hill et al^16^. reported similar findings that CCT was effective in improving working memory and verbal memory but did not detect a significant effect size in visual/ non-verbal memory as in our study. Such a discrepancy in visual memory is likely due to our updated inclusion of six additional studies (which accounts for half of our 11 included studies for visual memory) published from 2017 to 2023 after Hill et al.’s publication and the exclusion of one study due to the non-English main text. Our results are also consistent with another meta-analysis^17^ which demonstrated that CCT improves overall memory and working memory.

Also, our meta-analysis showed that CCT confers a benefit in people with dementia on verbal episodic memory, but not on visual and working memory. Several previous studies^16,20,21^ have reported cognitive training in general, computerized or not, was not efficacious in improving cognitive functions in dementia cohorts. In this meta-analysis, the positive efficacy on verbal memory in people with dementia was mainly driven by two recently published studies^22,23^ that found substantial improvements in memory function with supervised CCT and they recruited only participants with early stage AD. Hence, the positive efficacy detected in our meta-analysis can be attributed to the lower severity of dementia which may make them more responsive to cognitive training. Meanwhile, we note that more than half of the included studies on participants with dementia were at high risk of bias. We therefore recommend more clinical studies to be conducted to ascertain further the benefits of CCT among those diagnosed with dementia, especially those at the early stage.

Our meta-analysis reports the difference in efficacies between supervised and unsupervised CCTs in individuals with MCI. In different memory domains, supervised CCT demonstrated superior efficacies than unsupervised CCT. Such differences can be attributed to the face-to-face supervision results in better adherence, fewer distractions, and socializing opportunities for the participants. Notably, unsupervised CCT is only efficacious in improving verbal episodic memory, but not working or visual memory. Similarly, the effect sizes detected on verbal episodic memory are consistently greater than those detected in visual memory and working memory in both MCI and dementia cohorts in our meta-analyses. The better performance of verbal episodic memory observed in trained participants across various subgroups is consistent with several previous meta-analyses on general cognitive training in individuals with MCI, which also reported larger effect sizes in episodic memory than in working memory or nonverbal memory^24,25^.

Different subgroup analyses were performed to further investigate the potential confounding factors on the conclusions. Some previous literature^26,27^ suggested that the benefits of CCT might be limited to passive-controlled trials only. A more recent meta-analysis^16^ also reported that active-and passive-controlled trials gave comparable effect size estimates for CCT in MCI cohorts. Our results showed a greater effect in the passive-controlled trials than that in the active-controlled trials on verbal episodic memory, but no significant difference between the two types of controls on visual memory and working memory (Supplementary Table 3).

Nowadays, we have more well-proven cognitive assessment tools to screen for dementia and MCI^28^ and these screening tools are becoming increasingly accessible to the general public in different communities. With no pharmacological intervention for people with MCI, physicians would generally recommend regular exercise and cognitive training as the main part of a non-pharmacologic management strategy^5^. Traditionally, the mainstream cognitive training methods, including the computerized versions, are conducted under supervision by healthcare workers, but such a supervised approach is often resource-intensive. When more cases of cognitive impairment are identified due to the increasing accessibility of cognitive screening, the scarcity is even more exacerbated. Moreover, related healthcare manpower and resources are often wasted as the training is inappropriately matched with the population with different levels of cognitive impairment. Therefore, unsupervised or self-administered training, which is empowered by computerization and artificial intelligence, is an attractive option for broader implementation without requiring as many professional resources. In fact, experts have always been exploring whether unsupervised interventions can be as effective as supervised interventions. For example, a previous meta-analysis has found that unsupervised music therapy is more effective than interactive, supervised music therapy in relieving behavioral and psychological symptoms of people with dementia^29^. Our findings support supervised CCT as the preferred method to deliver the intervention as the effect sizes in verbal memory, visual memory and working memory are all significant and greater than those detected in unsupervised CCT. Nonetheless, despite showing greater benefits, supervised CCT requires both centralized facilities and trained professionals to deliver the intervention, which is not scalable due to resource limitations. Nowadays, it is easier to access both digital devices and internet connectivity, which helps to decentralize the implementation of CCT without face-to-face supervision. Hence, unsupervised CCT should be considered as a valuable alternative for broader implementation as it demonstrates benefit in improving verbal memory and it can be recommended to individuals with MCI who are waiting to be matched with supervised CCT resources.

This meta-analysis, conducted with stringent inclusion criteria and a reasonable sample size, demonstrates the efficacy of both supervised and unsupervised CCT on memory performance in people with MCI or at the early stage of dementia. There are still some limitations. First, studies only analyzed the immediate post-intervention effects of CCT and the short-term benefits of memory function with CCT were demonstrated in our analysis. Ongoing support for people with early symptoms of dementia is important. For long-term care, an unsupervised CCT with a lower manpower requirement should be promoted. Future studies should further explore the long-term benefits of CCT which may slow down the progression of MCI into dementia. Second, heterogeneity is observed across the included studies in all three memory domains in MCI, as reflected by the high I^2^ values. One of the main reasons may be the diversity of CCT interventions with different CCT designs, training durations and training frequencies. Such clinical heterogeneity cannot be eliminated, so random-effect models were used in the analysis to combine the evidence. Third, some eligible studies recruited both participants with MCI and participants with dementia and reported their results as a mixed cohort. Our exclusion of these mixed cohort studies would reduce the overall sample size of this meta-analysis, but it enhanced the similarity among the eligible studies. It also aligned with the primary focus of research that we aimed to study the benefits of CCT on individuals with MCI and those with dementia separately. Furthermore, training adherence is a potential limitation for the clinical application of CCT. None of the studies among the unsupervised CCTs reported poor compliance or low completion rates. Two studies even showed 100% completion rates with unsupervised CCT. Such a good adherence may be attributed to other remote supports, such as reminder messages to the participants or their family members. These confounding factors could not be totally adjusted, but it also highlights the importance of engagement strategies to enhance training adherence for unsupervised CCT. Finally, the potential publication bias always exists in systematic reviews, as unpublished studies can never be totally found in the literature search. To address potential publication bias, funnel plots’ asymmetries were assessed by Egger’s Test and the two detected asymmetric funnel plots were adjusted by Trim-and-fill methods. Both trim-and-fill adjusted effect size estimates remained statistically significant, despite slight reduction in magnitudes (Supplementary Table 4).

In this meta-analysis, CCT demonstrates improvements of memory functions in individuals with MCI; the benefit of unsupervised CCT for verbal memory function is also proven. Although CCT also showed benefits on verbal memory function in individuals with dementia, such results should be interpreted carefully because a high risk of bias is observed among the dementia studies. As the majority of the existing studies have focused on supervised CCT, future research directions should focus more on the effects of unsupervised CCT and investigate its potential in incorporating advanced technologies and artificial intelligence into self-administered cognitive training.

## Methods

This systematic review and meta-analysis adheres to the Preferred Reporting Items for Systematic Reviews and Meta-analyses (PRISMA) guidelines^30^ and was registered with PROSPERO under CRD42022363715 prospectively.

### Literature search

A literature search was performed in the electronic databases of MEDLINE, Embase and PsycINFO from inception to Sept 19, 2022, with keywords related to dementia/ MCI and CCT, including MCI, dementia, Alzheimer, computerized cognitive training, digital cognitive training, and web-based cognitive training (Supplementary Fig. 2). A supplementary search was conducted on Google Scholar on May 9, 2023 by the same keywords, in which the first 10 pages of all search records were screened. Inclusion criteria were: (1) participants were with MCI or dementia, either with diagnosis by clinicians or an established diagnostic criteria, such as the Petersen criterion^31^, the report of International Working Group on MCI^32^, the Clinical Dementia Rating scale^33^, the National Institute of Neurological and Communicative Disorders and Stroke and the AD and Related Disorders Association^34^, and The Diagnostic and Statistical Manual of Mental Disorders (DSM)^35^; (2) CCT interventions were used, with a structured training program of tasks to be completed by the participants through a computer or digital interface with an explicit goal to improve cognitive performance; (3) there was a non-CCT control group for comparison, examples of which include usual care, paper-and-pencil cognitive training, physical exercise, or unstructured cognitive activities (such as newspaper reading); (4) neuropsychological assessment scores of at least one memory domain from verbal episodic memory, visual memory and working memory were collected at baseline and immediately after the intervention period; and (5) only randomized controlled trials (RCT) were included. Exclusion criteria were: (1) Intervention programs that combined CCT with other types of non-CCT interventions in a multi-component fashion were excluded if the CCT component contributed less than 50% of the total training time; (2) Exergaming interventions, that incorporated aerobic exercise or vigorous physical activities into video games, were excluded; (3) Studies that specifically recruited people with concurrent mental disorders, brain injuries or AIDS were excluded; and (4) Studies published in languages other than English were also excluded.

### Data extraction

All study records from the literature search were independently screened by two reviewers (A.T.C.C. and J.Y.S.T.). The demographic details of individual trials as summarized in Table 1, including the year of publication, number of participants, mean age, gender distribution, types of interventions and control groups, were also extracted into a standardized Excel form. The outcomes were extracted and categorized into one of the three domains: (1) verbal episodic memory, (2) visual memory and (3) working memory by an established neuropsychological categorization method^36^ (Supplementary Table 5). Discrepancy records were resolved by the third reviewer (J.Y.C.C.).

### Interventions and outcome

CCT were classified into supervised and unsupervised subgroups. Supervised CCT is the traditional approach to administer CCT that requires supervision or the presence of trained healthcare professionals, such as occupational therapists or psychologists. It is usually conducted at scheduled time slots with centralized equipment in clinics or health centers. Unsupervised CCT is an emerging approach of self-administered CCT without real-time supervision or the presence of trained healthcare professionals or specialists. The training can be conducted with the users’ own computers or tablets at home or dwelling places without any time constraints. The benefits of CCT were separately evaluated on participants with MCI and those diagnosed with dementia. For the studies with a mixed cohort that included both types of participants, we sent emails to the primary authors to see if the data for the two types of participants could be separately retrieved and provided. Memory functions in the neuropsychological assessment scores were the main outcomes of this study. When a study reported multiple outcomes within the same memory domain, only one was selected in the ultimate analysis according to a pre-established priority list (Supplementary Fig. 3).

### Statistical analysis

Mean differences and standard deviations of the neuropsychological assessment scores on memory domain were extracted from each study. In consideration of the variation across different assessment scales, the standardized mean differences (SMD) were used in the meta-analysis. The heterogeneity was also assessed by I² to reflect the extent to which the variation across studies was mainly due to heterogeneity instead of random sampling error. As various CCT interventions were independently developed with different delivery methods and processes, the random-effects model was applied in the meta-analysis regardless of the significance of the heterogeneity levels. Review Manager (Version 5.4.1) was used to perform these meta-analyses to pool the SMDs from the CCT and control arms to estimate the effect size of CCT (with 95% Confidence Intervals). Egger’s Test of Intercepts and Trim-and-fill assessments were conducted in R version 4.2.2 by using the dmetar package^37^.

### Quality assessment

The GRADE method was used to assess the certainty of evidence based on five domains: risk of bias, imprecision, inconsistency, indirectness, and publication bias^38^. The certainty could be downgraded or upgraded depending on the quality assessment in each five domains. The resulting certainty of each finding could be “Very low”, “Low”, “Moderate” or “High”. Risk of bias was assessed according to Cochrane Collaboration’s Risk of Bias 2 tool^39^ guidelines in five domains: randomization process, deviations from intended interventions, missing outcome data, measurement of the outcome and selection of the reported result. If any domain was assessed to be at high risk, then the overall assessment of the study would be at “High Risk”. If any domain was assessed to raise some concerns but none of the other domains was at high risk, then the overall assessment would be “Some Concerns”. If all of the domains were rated at low risk, then the overall rating would be “Low Risk”. Publication bias was assessed by inspecting the asymmetry in funnel plots. For funnel plots that consisted of ten or more studies, asymmetry was further tested by Egger’s Test^40,41^. When significant asymmetry was detected by Egger’s Test, the Trim-and-fill technique would be used to adjust for the potential effect of publication bias^42^.

### Supplementary information

Supplementary Information

## Data Availability

Data collected and used in this meta-analysis can be requested from the corresponding author.
