# Supplementary material for: Computerized cognitive training for memory functions in mild cognitive impairment or dementia: a systematic review and meta-analysis
Source: NPJ Digit Med. 2024 Jan 3;7:1. doi: 10.1038/s41746-023-00987-5 (PMC10764827; doi:10.1038/s41746-023-00987-5)

Supplementary Information

Computerized Cognitive Training for Memory Functions in Mild Cognitive Impairment or Dementia: A Systematic Review and Meta-analysis

Supplementary Table 1: Risk of Bias Assessment……………………………………………….…………………….….2

Supplementary Table 2: Subgroup Analyses on Different Total Training Hours in Individuals with MCI ………….…….3

Supplementary Table 3: Subgroup Analyses on Different Types of Control in Individuals with MCI …………………....….3

Supplementary Figure 1: Overview of Adherence in Included Studies……………..……………………………………4

Supplementary Table 4: Funnel Plots…………………………………………………………..……………………..……5

Supplementary Figure 2: Search Strategy…………………………………………………….……………………..……..6

Supplementary Table 5: Descriptions of Memory Tests…………………………………………….…………………..…7

Supplementary Figure 3: Eligibility and Priority in Selecting Neuropsychological Assessment Score for Analysis.……...…8

Supplementary Figure 4: CCT on Memory Performance in Individuals with Dementia.………..……………..………………9

Supplementary Table 1: Risk of Bias Assessments

Study ID	Study Design	Cohort	D1	DS	D2	D3	D4	D5	Overall
Heiss 1994	RCT	Dementia	+	NA	!	+	-	!	-
Tarraga 2006	RCT	Dementia	+	NA	!	+	-	!	-
Barnes 2009	RCT	MCI	+	NA	+	+	+	!	!
Finn 2011	RCT	MCI	+	NA	+	+	+	!	!
Boller 2012	RCT	Dementia	+	NA	+	+	-	!	-
Herrera 2012	RCT	MCI	+	NA	+	+	+	!	!
Lee 2013	RCT	Dementia	+	NA	+	+	+	!	!
Fiatarone 2014	RCT	MCI	+	NA	+	+	+	+	+
Tarnanas 2014	RCT	MCI	+	NA	+	+	+	!	!
Finn 2015	RCT	MCI	+	NA	+	+	+	!	!
Barban 2016	Crossover RCT	MCI & Dementia*	+	!	!	+	-	!	-
Cavallo 2016	RCT	Dementia	+	NA	+	+	+	!	!
Gooding 2016	RCT	MCI	+	NA	+	+	-	!	-
Hyer 2016	RCT	MCI	+	NA	+	+	-	!	!
Lin 2016	RCT	MCI	+	NA	+	+	+	!	!
Hagovska_2017	RCT	MCI	+	NA	+	+	-	!	-
Han 2017	Crossover RCT	MCI	+	!	+	+	+	!	!
Savulich 2017	RCT	MCI	+	NA	+	+	+	!	!
De Luca 2018	RCT	Dementia	+	NA	+	+	+	!	!
Nousia 2018	RCT	Dementia	+	NA	+	+	-	!	-
Bernini 2019	RCT	MCI	+	NA	+	+	-	!	-
Li 2019	RCT	MCI	+	NA	+	+	+	+	+
Poptsi 2019	RCT	MCI	+	NA	+	+	+	!	!
Tang 2019	RCT	MCI	+	NA	+	+	+	+	+
Yang 2019	RCT	MCI	+	NA	+	+	+	+	+
Maneti 2020	RCT	MCI	+	NA	+	+	+	+	+
Park 2020	RCT	MCI	+	NA	+	+	-	!	-
Bernini 2021	RCT	MCI	+	NA	+	!	+	!	!
Callisaya 2021	RCT	MCI	+	NA	+	+	+	!	!
Kang 2021	RCT	MCI	+	NA	+	+	+	!	!
Nousia 2021	RCT	MCI	+	NA	+	+	+	!	!
Park 2022	RCT	MCI	+	NA	+	+	+	!	!
van Balkom 2022	RCT	MCI & Dementia*	+	NA	+	+	+	+	+
Yeh 2022	RCT	MCI	+	NA	+	+	+	+	+
Wu 2023	RCT	MCI	+	NA	+	!	+	!	!

+	Low risk
!	Some concerns
-	High risk
NA	Not Applicable
D1	Randomisation process
DS	Bias arising from period and carryover effects
D2	Deviations from the intended interventions
D3	Missing outcome data
D4	Measurement of the outcome
D5	Selection of the reported result

* denotes the studies that included both participants with MCI and those with dementia, and reported their results separately.

Abbreviations:

MCI=Mild Cognitive Impairment

RCT=Randomized Controlled Trial

Supplementary Table 2:
Subgroup Analyses on Different Total Training Hours in Individuals with MCI

	Verbal Episodic		Visual Episodic		Working Memory	
Total Training Hours	no. of Study	SMD (95%CI)	no. of Study	SMD (95%CI)	no. of Study	SMD (95%CI)
4-18 Hours	7	**0.40 (0.14-0.65)**	3	0.42 (-0.38-1.22)	8	0.24 (-0.11-0.59)
19-36 Hours	8	**0.63 (0.33-0.93)**	4	0.35 (-0.02-0.71)	4	0.48 (-0.19-1.14)
>36 Hours	6	**0.57 (0.04-1.10)**	3	**0.41 (0.04-0.78)**	3	0.55 (-0.29-1.39)

Abbreviations:
CCT=Computerized Cognitive Training, CI=Confidence Interval, MCI=Mild Cognitive Impairment, SMD=Standardized Mean Difference

Supplementary Table 3: Subgroup Analyses on Different Types of Control in Individuals with MCI

	Verbal Episodic		Visual Episodic		Working Memory	
Type of Control	no. of Study	SMD (95%CI)	no. of Study	SMD (95%CI)	no. of Study	SMD (95%CI)
Usual Care	11	**0.67 (0.33-1.01)**	6	**0.45 (0.11-0.80)**	7	0.29 (-0.13-0.72)
Active Control	11	**0.39 (0.20-0.58)**	5	0.26 (-0.10-0.62)	10	**0.43 (0.05-0.80)**

Abbreviations:
CCT=Computerized Cognitive Training, CI=Confidence Interval, MCI=Mild Cognitive Impairment, SMD=Standardized Mean Difference

Supplementary Figure 1. Overview of Adherence in Included Studies

Adherence of Supervised CCT in Individuals with MCI:

For the 19 studies that used supervised CCT on participants with MCI:

- Eight studies[27,39,41,43,46,48,49,50] did not report adherence data.
- Nine studies[24,28,29,31,32,34,36,52,53] reported full completion of interventions, except for the dropouts.
- One study[26] reported the mean completion rate of participants was 72.2% (i.e. 52 out of maximum 72 sessions).
- One study[45] reported the mean completion rate of participants was 91.6%.

Adherence of Unsupervised CCT in Individuals with MCI:

For the nine studies that used unsupervised CCT on participants with MCI, all studies reported adherence/ completion data. Also, all of them described their methods to ensure compliance of their participants who self-administered the CCT at home. A summary table of these unsupervised CCT are as follows:

Study	Adherence of Unsupervised CCT	Methods to Ensure Compliance
Barnes (2009)[21]	100% completion except for the drop-outs.	Subjects were contacted weekly to make sure they were progressing through the training. Progress was monitored weekly through automatically uploaded electronic data.
Finn (2011)[22]	Only participants with at least 80% completion were included in final assessment.	Adherence was monitored remotely on the training platform web site. All participants were also followed up with weekly telephone calls.
Lin (2016)[33]	The mean completion rate was 64.1% (i.e. 15.4 hours out of 24 hours maximum).	The completion % and score were recorded and reviewed on the system.
Han (2017)[35]	100% completion except for the drop-outs.	An occupational therapist delivered and picked up the tablet, without providing any assistance for using the application. The training records were uploaded automatically via a web portal service.
Li (2019)[40]	The average training duration was 122.8 min per week (out of 160 min max), i.e. 76.8%. The durations of all participants ranged from 100.0 (62.5%) to 158.6 min (99.1%).	Their login record and training performance on the web server were checked weekly, and telephone interview was conducted.
Tang (2019)[42]	All participants completed at least 90% of the training.	The progress was monitored by an independent neurologist through the training platform website. The neurologist would contact the participants or their families to remind them to complete the training if they missed any training.
Maneti (2020)[44]	Six participants completed 100% of the training. The remaining 12 participants completed more than 70%.	Individualized cognitive training exercises were reviewed and had the difficulty adjusted by the therapist once a week.
Callisaya (2021)[47]	Of the participants who did not drop out (n = 30), the average completion rate was 91.5%.	Feedback on adherence to participants was reviewed weekly by the researchers through the training app.
van Balkom (2022)[51]	The median completion of intervention was 100%, with all participants' completion rates ranging between 39% and 100%.	Intervention compliance was automatically registered and checked by researchers. They called the participants every two weeks to maximize involvement and solve potential issues.

Supplementary Table 4: Funnel Plots

<table>
<tr><th></th><th>Verbal Memory</th><th>Visual Memory</th><th>Working Memory</th></tr>
<tr>
<td>CCT (Overall) on People with MCI</td>
<td><u>Funnel Plot for Verbal Memory (MCI)</u>
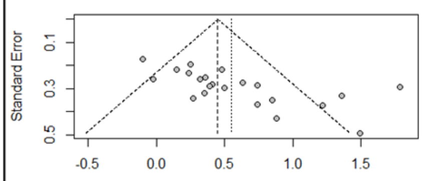
Egger's Test Result
Intercept 4.63
95% CI 2.45-6.82
Egger's Test p-value <0.001 (asymmetry detected)
<u>Trim-and-fill Adjustment</u>
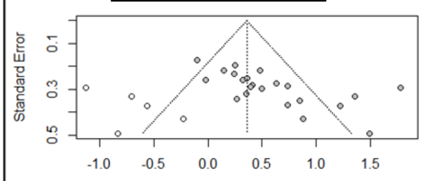
Effect Size before Trim-and-fill: 0.55 (0.35-0.74)
Effect Size after Trim-and-fill: 0.36 (0.10-0.62)</td>
<td><u>Funnel Plot for Visual Memory (MCI)</u>
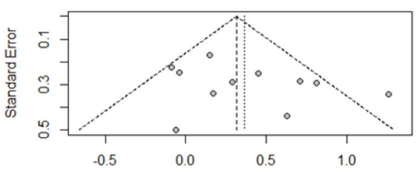
Egger's Test Result
Intercept 1.97
95% CI -0.97-4.91
Egger's Test p-value 0.22 (asymmetry not detected)</td>
<td><u>Funnel Plot for Working Memory (MCI)</u>
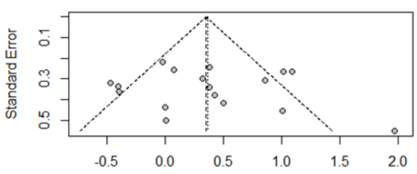
Egger's Test Result
Intercept 0.808
95% CI -2.69-4.31
Egger's Test p-value 0.66 (asymmetry not detected)</td>
</tr>
<tr>
<td>CCT (Supervised) on People with MCI</td>
<td><u>Funnel Plot for Verbal Memory (MCI)</u>
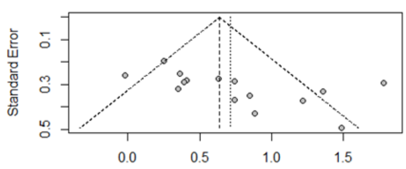
Egger's Test Result
Intercept 4.40
95% CI 1.05-7.75
Egger's Test p-value 0.02 (asymmetry detected)
<u>Trim-and-fill Adjustment</u>
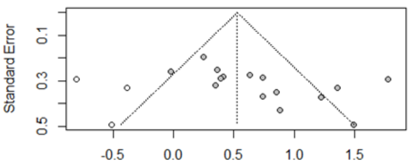
Effect Size before Trim-and-fill: 0.72 (0.45-0.98)
Effect Size after Trim-and-fill: 0.53 (0.19-0.87)</td>
<td><u>Funnel Plot for Visual Memory (MCI)</u>
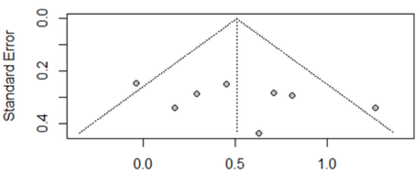
Egger's Test Result
Intercept 3.30
95% CI -2.34-8.94
Egger's Test p-value 0.3 (asymmetry not detected)</td>
<td><u>Funnel Plot for Working Memory (MCI)</u>
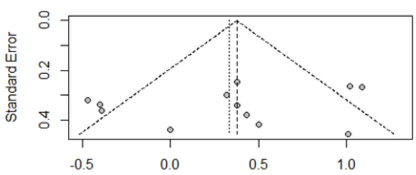
Egger's Test Result
Intercept -2.57
95% CI -7.65-2.51
Egger's Test p-value 0.35 (asymmetry not detected)</td>
</tr>
<tr>
<td>CCT (Unsupervised) on People with MCI</td>
<td><u>Funnel Plot for Verbal Memory (MCI)</u>
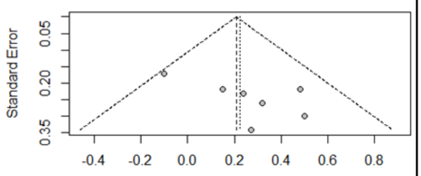
As number of studies is less than 10, funnel plot is visually inspected. Asymmetry is not detected.</td>
<td><u>Funnel Plot for Visual Memory (MCI)</u>
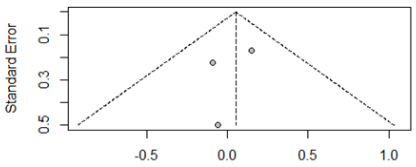
As number of studies is less than 10, funnel plot is visually inspected. Asymmetry is not detected.</td>
<td><u>Funnel Plot for Working Memory (MCI)</u>
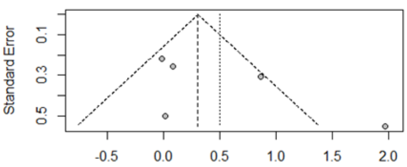
As number of studies is less than 10, funnel plot is visually inspected. Asymmetry is not detected.</td>
</tr>
<tr>
<td>CCT (Overall) on People with Dementia</td>
<td><u>Funnel Plot for Verbal Memory (Dementia)</u>
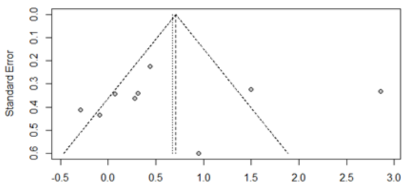
As number of studies is less than 10, funnel plot is visually inspected. Asymmetry is not detected.</td>
<td><u>Funnel Plot for Visual Memory (Dementia)</u>
As number of studies is only two, funnel plot is not plotted for assessment.</td>
<td><u>Funnel Plot for Working Memory (Dementia)</u>
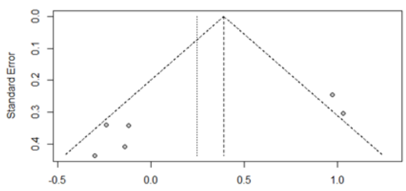
As number of studies is less than 10, funnel plot is visually inspected. Asymmetry is not detected.</td>
</tr>
</table>

Supplementary Figure 2: Search Strategy

OVID + PubMed databases: Medline, Embase, PsycINFO

Search conducted on Sept 19, 2022

Search Strategy:

1 (Mild cognitive impairment or MCI or memory impair* or cognitive impair* or subjective memory impair* or subjective memory disorder? or dement* or Alzheimer*).ab.

2 (computer* cognitive or virtual reality or augment* reality or web based cognitive or web-based cognitive or app based cognitive or app-based cognitive or digital* cognitive or computer* memory or computer* memory or web-based memory or web based memory or app-based memory or app based memory or digital* memory).ab.

1+2

Supplementary Search Strategy by Google Scholar
Search conducted on May 9, 2023

First Search Strategy:

(mild cognitive impair OR MCI OR memory impair OR cognitive impair OR subjective memory impair OR subjective memory disorder OR dement OR Alzheimer)

AND

((computer AND “cognitive”) OR “virtual reality” OR (augment AND “reality”) OR (web based cognitive) OR (app based cognitive) OR (digital cognitive) OR (computer memory) OR (web based memory) OR (app based memory) OR (digital memory))

Second Search Strategy:

CCT RCT MCI OR Dementia

Supplementary Table 5: Descriptions of Memory Tests

Types of Memory Tests	Descriptions	Examples of Neuropsychological Tests
**Verbal Episodic Memory**	Conscious recollection of long-term, explicit memory of previous episodes of **verbal nature**. Test methods include **word list recall and story recall**.	1. Rey Auditory Verbal Learning Test (RAVLT) - Words Delayed Recall<br>2. Wechsler Memory Scale 3rd Edition (WMS-III) - Word List Delayed Recall<br>3. WHO-UCLA Auditory Verbal Learning Test - Delayed Recall<br>4. Rivermead Behavioral Memory Test (RBMT) - Story Delayed Recall<br>5. Wechsler Memory Scale 3rd Edition (WMS-III) - Logical Memory Subset II
**Visual Episodic Memory**	Conscious recollection of long-term, explicit memory of previous episodes of **non-verbal, visual nature**. Test methods include **visual production and visual recognition**.	1. Rey Complex Figure Recall Test<br>2. Wechsler Memory Scale 3rd Edition (WMS-III)- Visual Reproduction Subset<br>3. Rivermead Behavioral Memory Test (RBMT) – Picture Recognition
**Working Memory**	**Immediate memory** of a limited-capacity store for retaining short-term information (often seconds to 1-2 minutes) for performing mental operations. Test methods include **digit or spatial span tests**.	1. Digit Span Forward Test<br>2. Digit Span Backward Test<br>3. Digit Span Forward and Backward Test<br>4. Spatial Span Test<br>5. Block Tapping Test

Supplementary Figure 3:
Eligibility and Priority in Selecting Neuropsychological Assessment Score for Analysis

Verbal Episodic Memory

When multiple episodic memory outcomes were available in a study, one was selected according to the priority list as follows (in descending order from most preferred to least preferred):

i. Word list recall,
ii. Logical Memory Test,
iii. Story Recall, or
iv. Mixture of above or composite score of verbal episodic memory.

The verbal episodic memory outcomes reported by the included studies involved immediate recall or delay recall duration ranging from 5 to 30 minutes. When a specific episodic memory assessment was conducted and reported with results at multiple delay intervals, the data of the longest delay interval was selected.

Visual (Non-verbal Episodic Memory)

The visual memory tests included the following types of neuropsychological assessments and were selected according to the priority list as follows:

i. Figure/ Pattern Reproduction by Drawing, or
ii. Visual Recognition.

The visual memory outcomes reported by the included studies involved immediate recall or delay recall duration ranging from 3 to 30 minutes. When a specific visual memory assessment was conducted and reported with results at multiple delay intervals, the data of the longest delay interval was selected.

Working Memory

When multiple working memory outcomes were reported, one was selected according to the priority list as follows (in descending order from most preferred to least preferred):

i. Digit Span Forward,
ii. Digit Span Backward,
iii. Digit Span Forward and Backward,
iv. Digit Span in Unspecified Direction,
v. Spatial Span Test,
vi. Block Tapping Test,
vii. Letter Number sequencing Test,
viii. Dot Counting Test,
ix. 1-back Test, or
x. Mixture or above, or composite score of working memory.

Supplementary Figure 4: CCT on Memory Performance in Individuals with Dementia

Study or Subgroup	CCT Mean	CCT SD	CCT Total	Non-CCT Mean	Non-CCT SD	Non-CCT Total	Weight	Std. Mean Difference IV, Random, 95% CI
**Verbal Episodic Memory**								
Heiss 1994	0.41	1.041	18	0.09	1.077	17	11.4%	0.30 [-0.37, 0.96]
Tarraga 2006	0.4	1.007	15	0.12	1.006	16	11.2%	0.27 [-0.44, 0.98]
Boller 2012	-0.5	1.701	12	-0.08	1.383	12	10.8%	-0.26 [-1.07, 0.54]
Lee 2013	4.17	3.633	7	1.29	1.914	6	9.0%	0.90 [-0.27, 2.07]
Barban 2016	0.4	1.266	42	-0.1	1.201	39	12.3%	0.40 [-0.04, 0.84]
Cavallo 2016	1	1.181	38	-2	1.08	38	11.6%	2.62 [2.00, 3.25]
De Luca 2018	0.6	2.774	20	0.4	3.153	15	11.4%	0.07 [-0.60, 0.74]
Nousia 2018	1.04	1.122	25	-0.28	0.485	25	11.6%	1.50 [0.87, 2.14]
van Balkom 2022	0.333	3.937	9	0.615	2.501	13	10.6%	-0.09 [-0.94, 0.76]
**Subtotal (95% CI)**			**186**			**181**	**100.0%**	**0.64 [0.02, 1.27]**
Heterogeneity: Tau² = 0.77; Chi² = 59.96, df = 8 (P < 0.00001); I² = 87%								
Test for overall effect: Z = 2.02 (P = 0.04)								
**Visual Memory**								
Boller 2012	2.5	4.821	12	-0.5	4.373	12	51.6%	0.63 [-0.19, 1.45]
van Balkom 2022	0	4.899	9	-0.231	2.127	13	48.4%	0.06 [-0.79, 0.91]
**Subtotal (95% CI)**			**21**			**25**	**100.0%**	**0.36 [-0.24, 0.95]**
Heterogeneity: Tau² = 0.00; Chi² = 0.88, df = 1 (P = 0.35); I² = 0%								
Test for overall effect: Z = 1.18 (P = 0.24)								
**Working Memory**								
Heiss 1994	-0.64	1.072	18	-0.03	3.33	17	16.8%	-0.24 [-0.91, 0.42]
Boller 2012	0.16	0.607	12	0.25	0.673	12	14.9%	-0.14 [-0.94, 0.67]
Cavallo 2016	1.1	1.175	38	-0.02	1.256	38	19.5%	0.91 [0.44, 1.39]
De Luca 2018	0.5	1.092	20	0.6	0.772	15	16.7%	-0.10 [-0.77, 0.57]
Nousia 2018	1.12	1.327	25	-0.16	1.122	25	17.8%	1.03 [0.43, 1.62]
van Balkom 2022	-0.556	1.59	9	-0.154	1.068	13	14.2%	-0.30 [-1.15, 0.56]
**Subtotal (95% CI)**			**122**			**120**	**100.0%**	**0.24 [-0.28, 0.76]**
Heterogeneity: Tau² = 0.30; Chi² = 18.75, df = 5 (P = 0.002); I² = 73%								
Test for overall effect: Z = 0.90 (P = 0.37)								

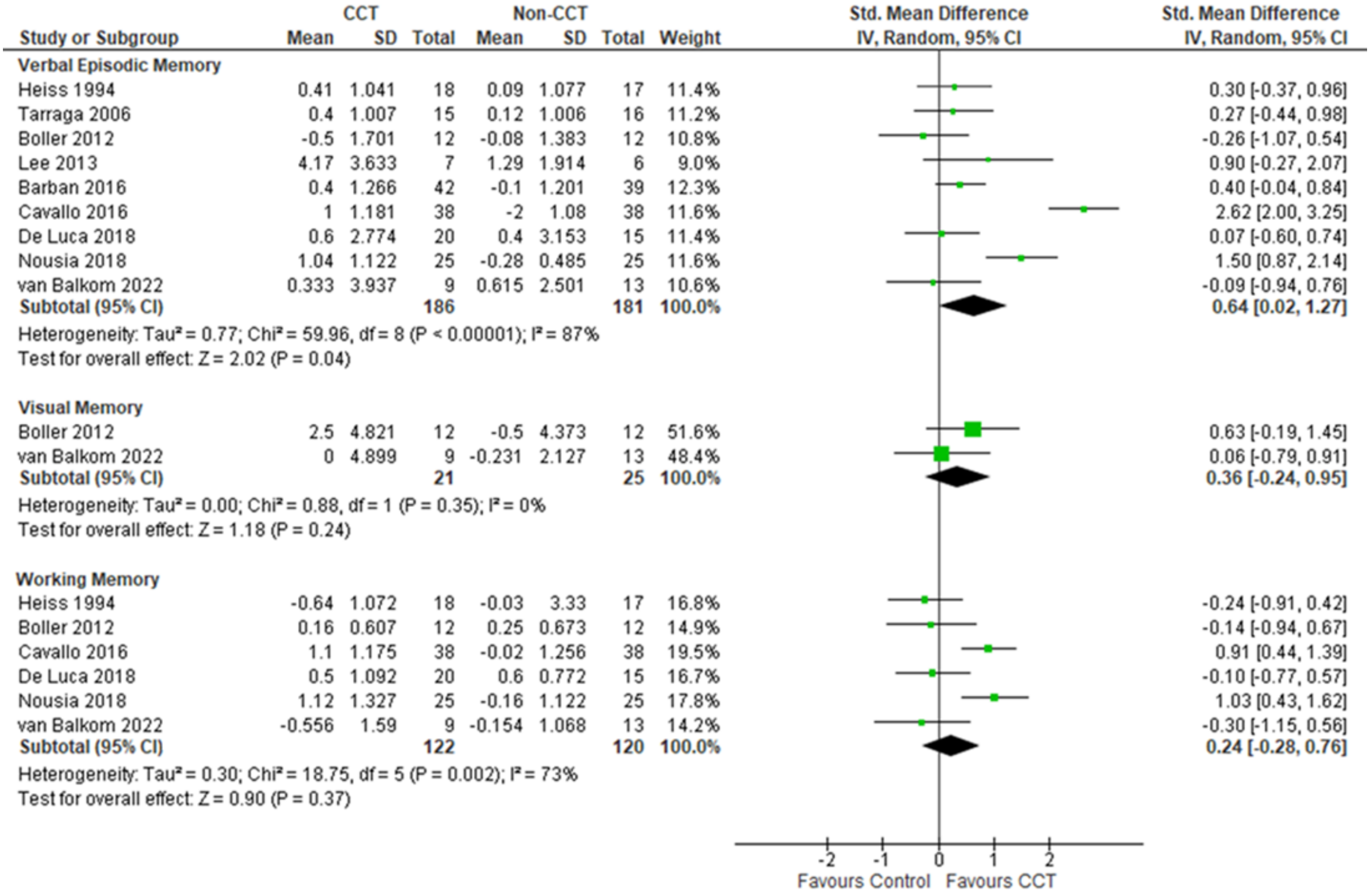

Supplement: Supplementary file 1 — Supplementary Information [file 41746_2023_987_MOESM1_ESM.pdf]
